# *Dirofilaria repens* in a Pediatric Patient—First Case Report from Switzerland

**DOI:** 10.1055/s-0043-1768706

**Published:** 2023-05-14

**Authors:** Rebekka Rose, Kai-Uwe Kleitsch, Diana Born, Pascal Heye

**Affiliations:** 1 Clinic for Pediatric Surgery, Children's Hospital of Eastern Switzerland, St. Gallen, Switzerland; 2 Institute of Pathology, Cantonal Hospital St. Gallen, St. Gallen, Switzerland

**Keywords:** Dirofilaria, *D. repens*, spermatic cord, testis, scrotum

## Abstract

We report the first case of *Dirofilaria repens* in a 4-year-old male patient in Switzerland. The disease is a vector-borne parasitic infection that is not endemic to Switzerland. A 4-year-old male presented with a tender mass in the left groin. The patient was taken to the operating room for surgical exploration to rule out a pathology that could be harmful to the spermatic cord. A node was found along the spermatic cord and excised. Histopathology and microbiology studies revealed the diagnosis of *Dirofilaria repens* .

Even though Switzerland is not endemic to *Dirofilaria repens* , the diagnosis of a parasitic infection should be considered in patients presenting with subcutaneous nodules in correlation with a travel history to endemic areas. The treatment consists of complete excision of the affected tissue.

## Introduction

*Dirofilaria repens* ( *D. repens* ) belongs to the species of filarial nematodes. Their primary hosts are dogs, foxes, and other carnivores. 1 2 3 In primary hosts, the larvae, called microfilariae, are small enough to spread through the host's body, mature to a fertile state and continue to multiply. The vectors for a zoonotic infection are mosquitoes. In rare cases, humans may accidentally get infected by the sting of an infected mosquito, at which point the incubation period is 6 to 8 months. 4 Since nematodes of *D. repens* cannot reach the adult stage of their lifecycle in humans, they remain confined to or near the local site of infection, without the risk of a systemic infection. Humans most commonly present with subcutaneous nodes that may be accompanied by local edema, overlying redness, rash, or pain. Lesions may appear anywhere in the body and have been reported in the skin, eyes, lungs, and viscera. *D. repens* is mainly endemic in the Mediterranean and eastern countries of Europe, Africa, and Asia. 2 3 4 Given the changing global environment (international tourism, animal trade, and global warming) and an increasing habitat for mosquitoes, the parasites' endemic area has become larger. 1 2 3 4 Only a few reports are available on Dirofilariasis in the male genitalia in children, and none so far in Switzerland. We report the first case of *D. repens* infection in a pediatric patient in Switzerland.

## Case Report

### Patient History and Exam

A 4-year-old male patient was referred to the emergency department (ED) for a tender lump in his left groin. The lesion was present for 3 months, however, acute pain and swelling only started 24 hours prior to presentation. Upon clinical exam, the patient was afebrile with normal vital signs. An approximately 1 × 1 cm tender mass was palpated in his left groin along the spermatic cord proximal to the external inguinal ring. The overlying skin was intact, with local redness and edema. The scrotum was mildly swollen and red, with a normal clinical exam of both testicles. Initially, the ED considered the diagnosis of an inguinal hernia and attempted a reduction. It was found that the mass could not be reduced. An ultrasound of the groin demonstrated edema and hyperperfusion along the spermatic cord and epididymis, consistent with funiculitis and epididymitis. There was a small hydrocele and lack of any signs suggestive for testicular torsion. Blood work demonstrated a normal leucocyte count, normal levels of eosinophil, and increased levels of neutrophil granulocytes, along with mild lymphopenia. The patient was otherwise healthy. The family lived in a rural setting and had a dog. A travel history was positive for travelling to Croatia 4 months prior to the first presentation of the node. At that time, the patient had been stung by mosquitoes multiple times.

### Surgical Treatment

**Fig. 1:**
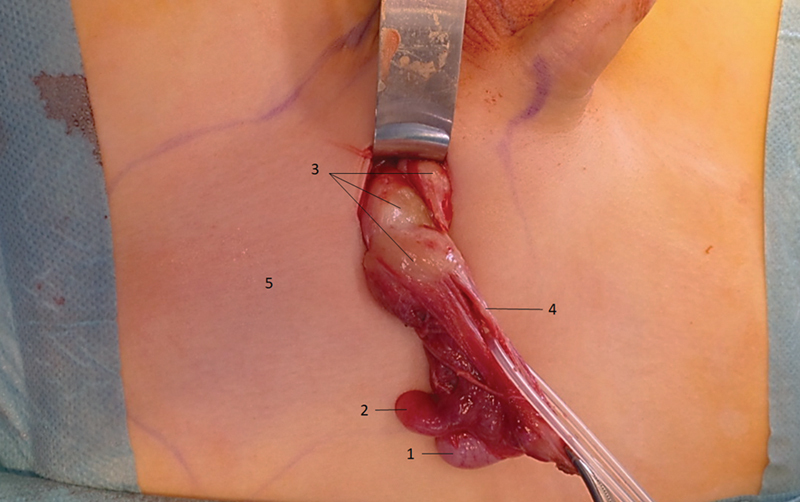
Surgical exploration of the left groin demonstrating the testicle (1), the epididymis (2), soft tissue nodules (3), the spermatic duct (4), and skin erythema (5).

After initial presentation in the ED, the patient was discharged home with symptomatic treatment for epididymitis. He returned within 12 hours with progressive pain and soft tissue edema. Due to progressive symptoms, the patient was taken to the operating room for surgical exploration to rule out a pathology that could be harmful to the spermatic cord. Following an inguinal incision, the subcutaneous tissue was found to be inflamed and edematous. The external oblique fascia was opened and the testis along with the spermatic cord was delivered. An encapsulated hydrocele along the spermatic cord was opened and drained amber-colored serous fluid. The testis and epididymis appeared inflamed but with otherwise normal anatomy. An indurated soft tissue nodule was found adherent to the internal spermatic fascia along the spermatic cord ( Fig. 1 ). The mass was dissected off the spermatic duct and vessels and sent for fresh frozen section. The pathology demonstrated an inflammation with abundant eosinophil granulocytes of possible parasitic origin, with no signs of malignancy. Subsequently, the procedure was concluded by ligation of the processus vaginalis and orchidopexy.

After consulting infectious disease, no additional medical treatment was given. The postoperative course was uneventful.

### Pathology and Microbiology

**Fig. 2:**
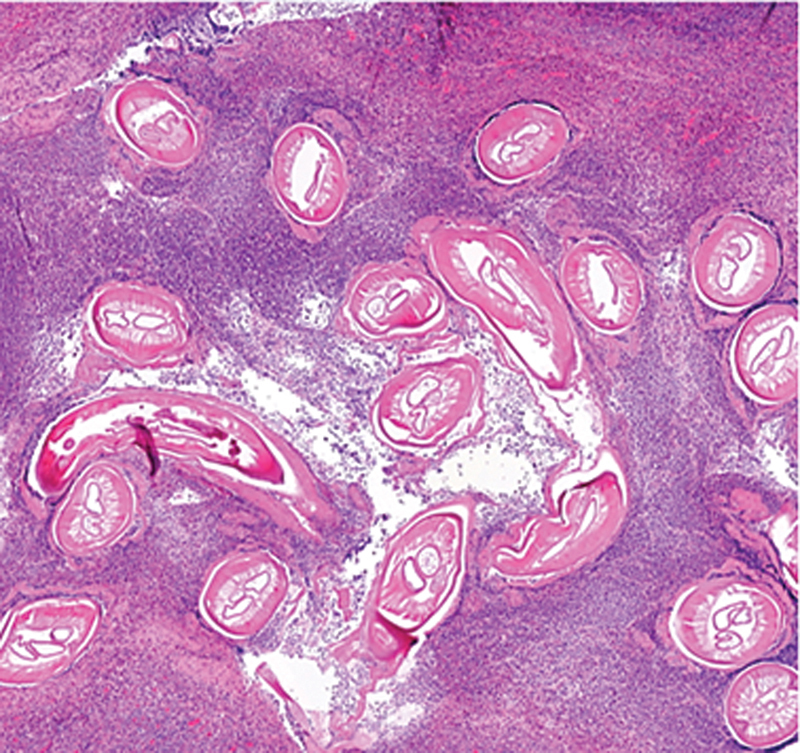
Microscopic slide with hematoxylin and eosin stain demonstrating multiple sections of filarial nematodes, surrounded by eosinophil granulocytes.

The definitive histopathology of the specimen with paraffin embedding and hematoxylin and eosin staining demonstrated connective tissue with parts of a filarial nematodes ( Fig. 2 ). It was accompanied by extensive soft tissue inflammation with eosinophil invasion and destruction of surrounding skeletal muscle. The diagnosis was confirmed by polymerase chain reaction, revealing a more than 99% match for *D. repens* . Microbiology swaps of the hydrocele, the testis, and the lesion returned negative.

## Discussion

This is the first report from Switzerland of an infection with *D. repens* along the spermatic cord. To screen for similar reports of *D. repens* in male patients, a literature review was performed using combinations of *D. repens* and spermatic cord, testis, epididymis, and scrotum in PubMed. The search yielded 20 case reports, with six including children (median age: 11 years, range: 11 months to 13 years). 5 6 7 8 9 10

In the majority of cases, the pathology developed over time, commonly presenting as a solid or cystic mass. Only a few cases presented acutely, mimicking acute scrotum, testicular torsion, or incarcerated inguinal hernia. Our patient had traveled to Croatia 4 months prior to the first presentation of the node, which he had for 3 months before presenting to the ED with acute symptoms. This is consistent with previously reported symptoms, and with an incubation period of 6 to 8 months. 4

Diagnostic blood work and ultrasound generally demonstrated similar results to our case, with oval nodules with cystic contents in selected cases. On rare occasions, radiologists were able to demonstrate linear hyperechogenic structures, sometimes with spontaneous movement, representing worms. Intraoperative localization of the pathologies was described along the spermatic cord, in and on the testis and epididymis, and in the subcutaneous tissue of the scrotum. The tissue was reported as firm nodules, cysts, or mixed solid-cystic lesions. In all cases, resection of the pathology was the treatment of choice. Knowledge of this entity is essential to the pediatric surgeon, as some surgeons performed hasty orchiectomy, including the case of a 13-year-old boy with preoperative concern for testicular malignancy. 8 In all cases, the diagnosis of a parasitic infection with *D. repens* was made after histopathological examination of the extirpated tissue.

## Conclusion

As international traveling continues to grow and the natural habitat of mosquitoes expands, *D. repens* is becoming more relevant in central Europe. It should be considered in patients presenting with subcutaneous nodules in correlation with a travel history to endemic areas. Knowledge of this disease is essential for timely diagnosis and correct surgical treatment.

## Notes

New Insights and the Importance for the Pediatric Surgeon
Pediatric patients commonly present with inguinal masses, which may be accompanied by local or systemic signs of infection. With *Dirofilaria repens* , we present an uncommon differential diagnosis, which may, however, become more common in the future, given the growing international traveling and the expansion of the habitat of mosquitoes.
